# Importance of digital skills in attitude towards the use of smartphones and usage behavior in ederly population

**DOI:** 10.1002/alz.092986

**Published:** 2025-01-09

**Authors:** Estela Benitez, Elsa Araceli Revollo Sarmiento, Maria Celeste López Moreno, Rocio Micaela Vera, Leticia Vivas

**Affiliations:** ^1^ Universidad Nacional Mar del Plata, Mar del plata, Buenos Aires Argentina; ^2^ IPSIBAT (UNMDP‐CONICET), Mar del Plata, Buenos Aires Argentina; ^3^ IPSIBAT (UNMDP/CONICET), Mar del Plata, Buenos Aires Argentina; ^4^ Universidad Nacional Mar del Plata, mar del plata, Buenos Aires Argentina; ^5^ IPSIBAT (CONICET/National University of Mar del Plata), Mar del Plata, Buenos Aires Argentina

## Abstract

**Background:**

The use of technology in elderly population has increased in the past years due to COVID‐19. Therefore it is relevant to identify the factors that contribute and limit the effective use of technology by older people. In particular, digital skills have shown to be relevant to determine the attitude toward the use of technology and consequently to have an effect on usage behavior. The aim of the current poster is to analyze the relations between this variable and the use of smartphones.

**Method:**

We employed a correlational design with a non‐probabilistic for convenience sample including 139 elderly (56.9% women). The age ranged from 60 to 90 years, with a mean of 71.1 (DS = 6.62). Participants were administered the Digital Competencies Questionnaire (Heredia & Garcia, 2017), the acceptance questionnaire for the STAM model smartphone (Chen & Chan, 2014) and questionnaire to assess usage behavior called SAMCOP (Ma et al., 2016)

**Result:**

First, a correlation analysis was performed between digital competencies (DC) and the variables of the STAM and it indicated a positive correlation between DC and self‐efficacy in use (r = .456; p < .001), facilitating conditions (r = .434; p < .001), perceived usefulness in use(r = .508; p < .001), perceived ease of use (r = .425; p < .001), attitude towards use (r = .594; p < .001), and a negative correlation with anxiety towards use with the use of technology (r = ‐.323; p < .01). Furthermore, a linear regression analysis was performed to identify the influence of DC on SAMCOP. The results indicated an influence of DC on frequency of smartphone use (p < .001; R2 = .245); experience in smartphone use(p < .001; R2 = .175); and most frequently used apps (p < .01; R2 = .0801), but no influence on number of installed apps (p = .491).

**Conclusion:**

The results demonstrate a direct relationship between digital competencies and smartphone acceptance variables. This indicates that greater digital competencies lead to higher smartphone acceptance and reduced anxiety in the elderly. These competencies also influence smartphone usage behavior in old age, highlighting the importance of promoting digital skills among the elderly.

